# Frequencies of Blood Group Systems MNS, Diego, and Duffy and Clinical Phases of Carrion's Disease in Amazonas, Peru

**DOI:** 10.1155/2014/576107

**Published:** 2014-03-31

**Authors:** Oscar Acosta, Luis Solano, Jorge Escobar, Miguel Fernandez, Carlos Solano, Ricardo Fujita

**Affiliations:** ^1^Institute of Tropical Medicine Daniel A. Carrion, Faculty of Medicine, Major National University of San Marcos, Lima, Peru; ^2^Genetics and Molecular Biology Center, Faculty of Medicine, University of San Martin of Porres, Lima, Peru; ^3^Institute of Biological Chemistry, Microbiology and Biotechnology, Faculty of Pharmacy and Biochemistry, Major National University of San Marcos, Lima, Peru; ^4^Peruvian Ministry of Health (MINSA), Direction of the Sub Region of Health Bagua, Bagua Grande, Amazonas, Peru; ^5^Peruvian Ministry of Health (MINSA), General Direction of Environmental Health (DIGESA), Lima, Peru

## Abstract

Carrion's disease (CD), is a human bartonellosis, that is, endemic in the Andes of Peru, Ecuador, and Colombia. *Bartonella bacilliformis*, a native hemotrophic bacteria, is the causative agent of CD, and the interaction with the host could have produced changes in the gene frequencies of erythrocyte antigens. The goal here is to investigate the relationship between allele frequencies of blood group systems MNS, Diego, and Duffy and the clinical phases of CD, within a genetic context. In this associative and analytical study, 76 individuals from Bagua Grande, the province of Utcubamba, and the department of Amazonas in Peru, were enrolled. Forty of them resided in Tomocho-Collicate-Vista Hermosa area (high prevalence of cases in chronic phase, verrucous, or eruptive phase, without previous acute phase). Thirty-six individuals were from the area of Miraflores (high prevalence of cases in acute phase only) and were evaluated for blood group systems MNS, Diego, and Duffy. This study constitutes one of the first attempts at evaluating the genetic factors and clinical phases of CD. No significant statistical differences (*P* > 0.05) between allele frequencies of blood groups MNS, Diego, and Duffy and the prevalence of chronic and acute phases were detected in the two areas of Amazonas, Peru.

## 1. Introduction

**Figure 1:**
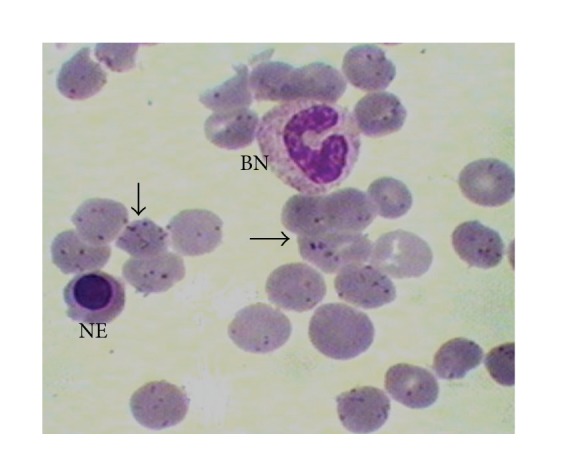
Blood smear of patient in acute phase. Parasitized erythrocytes by bacillary and coccoid forms of* B. bacilliformis *(arrows), a nucleated erythrocyte (NE), and a band neutrophil (BN) can be observed. Giemsa stain, 1000x.

Carrion's disease (CD) is a human bartonellosis, an infectious pathology caused by* Bartonella bacilliformis* (*B. bacilliformis*), a bacteria native to the Andes, which is able to enter the bloodstream and parasitically invade erythrocytes (Figure 1). It is transmitted to humans through the bite of hematophagous insects of the genus* Lutzomyia*, mainly the species* L. verrucarum* [1, 2]. At present, it is endemic in the Andes (between 500 m and 3200 meters above sea level) and the coastal and Amazonian and inter-Andean valleys of Peru, Ecuador, and Colombia. However, it has recently been found to be spreading and emerging in lower ecosystems in some coastal and Amazonian locations. In Peru, human bartonellosis has been found in different regions along the Andes such as Piura, Amazonas, Cajamarca, La Libertad, Ancash, Lima, Huancavelica, Ayacucho, and Cusco [3, 4].

The Carrion disease has been divided in to several clinical phases; two of the most remarkable are as follows: (1) the hematic or acute phase, known as “Oroya fever,” common symptoms of which are fever, hemolytic anemia, cephalea, pallor, myalgias, artharlgias, and loss of consciousness and (2) the eruptive or verrucous phase, termed “verruga peruana” (Peruvian wart), which appears after the recovery from the acute phase, reddish verrucous eruptive lesions of different sizes and shapes characterize this phase, in some cases, patients without a history of the acute illness can display this phase [4, 5].

In endemic areas, the presence of asymptomatic carriers is important; research so far has indicated humans to be the only reservoir of the bacterium. Evidence suggests that outbreaks of bartonellosis represent the emergence, in areas of nonendemicity, or the resurgence of the disease, which appears to be an infectious emerging or reemerging pathology with a wide geographic range [4, 6].

Several studies [1, 5, 7] have been conducted with the clinical aspects, traditional epidemiology, geographical distribution, and molecular biology of the bacterium and other topics related to* B. bacilliformis*. Epidemiological data shows that in some closely situated Peruvian areas, differences in the predominance of the CD phases can be noted. Comparison of the clinical phases in localities within the district of Bagua Grande, Amazonas department, showed different distributions of either the verrucous or hematic phases. In Tomocho-Collicate-Vista Hermosa there was a prevalence of verrucous cases (81 affected individuals versus 5 hematic occurrences), whereas, in the nearby location of Miraflores, there were 12 hematic cases and only 5 verrucous affected individuals. This information was obtained by MF and JE (data not published) and Records of the Epidemiological Week N° 32, August 2004 (provided by Epidemiological Office, Subregion of Bagua-Amazonas, Ministry of Health).

The local population is apparently mestizo (mostly Andean and Caucasian genetic background), most of whom are migrants coming from the Andean department of Cajamarca (communication of Subregion of Bagua-Amazonas).

Genetic factors can exert a great influence on the susceptibility, resistance, and/or tolerance to infectious disease, not only at an individual but also at a population level. It is well known that some genetic traits considered of risk are present in modern human populations and that they arise independently due to selective pressure of infections. Thus, mutant globins, glucose-6-phosphate dehydrogenase (G6PD), and Duffy antigen genes are paradigmatic examples of malaria protection. An abnormally high prevalence of deleterious mutant alleles is present in populations of the Tropical Belt areas around the world. For example, the enzyme G6PD is involved in malaria protection but its homozygous state predisposes to hemolytic anemia. People with an absent Duffy antigen are also protected against malaria by* Plasmodium vivax *(*P. vivax*) because the pathogen uses it to invade erythrocytes [8–11].

Very little information is available from South American populations about this aspect. There are native parasites that cause endemic diseases since pre-Columbian times such as* Trypanosoma cruzi*, Leishmania peruviana, and* B. bacilliformis* [12, 13] and it is possible that they have influenced the distribution of different alleles by selective pressure. Although the malaria is important, the pre-Columbian presence of* P. vivax* has been subject to great debate and remains unclear [14].

We postulate that Amerindian hosts have possibly developed mechanisms of susceptibility, resistance, or tolerance with regard to genetic composition which may be associated with the clinical phases of CD. One strategy to test this hypothesis is to search for genes or molecular markers in which selective pressure can be exerted by* B. bacilliformis* and to evaluate the variation of allele frequencies of the genes in human populations.

Specifically, this can be verified by variation of erythrocyte antigens (receptor molecules for the bacterium) and cellular and humoral immunity. According to Buckles and McGinnis [15], glycophorins A/B and band 3 act as receptors for* B. bacilliformis* adhesion and invasion molecules such as flagellin, adhesin, deformin, IaIA, and IaIB. Glycophorins A/B and band 3 are closely related with blood group systems MNS and Diego [16]. On the other hand, Duffy group is associated with resistance to malaria by* P. vivax* in populations living in endemic areas in Africa and Oceania [17, 18]. It is possible that, in Bartonella infection, Duffy glycoproteins are involved as receptor molecules. Thus, variability or polymorphisms of blood group systems could be related with susceptibility, resistance, and/or tolerance to infection, as well as with the prevalence of the disease's clinical phases.

In this study, genetic and coevolutionary principles are applied in order to investigate the relationship between the genetics of Amerindian host and clinical phases of CD, an infectious pathology that can be found in most of the Andean departments of Peru.

Specifically, the goal is to investigate the relationship between clinical phases of CD and the allele frequencies of blood group systems MN, Ss, Diego, and Duffy under a genetic coevolutionary context, that is, under selective pressure of* B. bacilliformis* over the Amerindian host. This research is one of the first attempts to evaluate genetic factors and clinical phases of this ancient disease from the Andes. For this purpose, we have taken advantage of the differences between Tomocho-Collicate-Vista Hermosa (predominance of verrucous phase) and Miraflores (predominance of hematic disease). We will compare the allele frequencies of the blood group systems MN, Ss, Diego, and Duffy between these two areas.

## 2. Materials and Methods

### 2.1. Areas of Study and Participants

**Figure 2:**
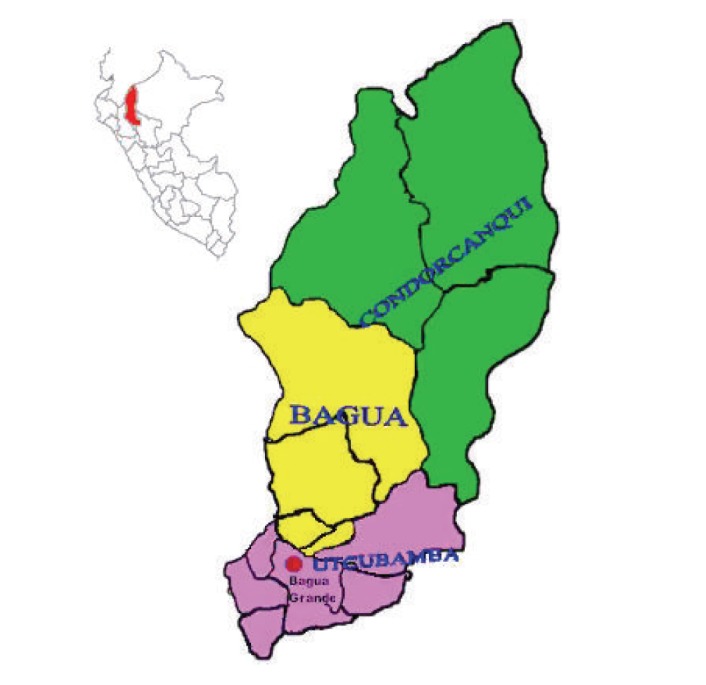
Location of the sampled areas in Bagua Grande (red spot), province of Utcubamba, department of Amazonas, Peru. At left top, map of Peru with location of department (filled in red).

CD is endemic within the area of study corresponding to the district of Bagua Grande (05°46′40′′ South, 78°25′46′′ West), province of Utcubamba, department of Amazonas, in the Northern Upper Amazonian Jungle of Peru at 540 meters above sea level (Figure 2). We have analyzed samples at two locations with different prevalence of either the verrucose or hematic forms according to the Annual Report and Epidemiologic Bulletin Subregion of Health Bagua-Amazonas, Peru, 2004. General and clinical data was collected for each of the 40 individuals from Tomocho-Collicate-Vista Hermosa localities (prevalence verrucose form or chronic phase) and for the 36 from Miraflores district (prevalence hematic form or acute phase), using convenience sampling.

### 2.2. Biological Sample

The sampling was performed in September 2004 (Epidemiological Week no. 36). Each of the 76 individuals signed a written consent form before samples were collected. Five to 10 mL of peripheral blood were collected in Vacutainer sterile tubes (Becton Dickinson, Franklin Lakes, NJ) containing anticoagulant EDTA or sodium citrate. The samples were kept under refrigeration until they were processed for blood groups at the Laboratory for Bartonellosis, Daniel A. Carrion Tropical Medicine Institute, Universidad Nacional Mayor de San Marcos (UNMSM). This study was presented to and approved by the Scientific and Ethic Committee Faculty of Medicine, UNMSM. In addition, the project was approved and authorized by the Subregional Director of Health in Amazonas and Bagua's Hospital.

### 2.3. Determination of Blood Groups

Blood typing was performed according to antisera manufacturer's specifications (Immucor Gamma Biological, USA): group MN was determined by monoclonal anti-M and anti-N antisera through a direct method in test tubes. While blood groups Ss, Diego, and Duffy were determined by polyclonal anti-S, anti-s, anti-Dia, anti-Fya, and anti-Fyb antisera through Human Antiglobulin test and test tube method.

### 2.4. Statistical Analysis

Allele frequencies, based on phenotype frequencies, were obtained by a direct counting method. In order to evaluate the frequencies, a Chi-square test (*χ* ^2^) was used to estimate the Hardy-Weinberg equilibrium. A *χ* ^2^ or Fisher's exact test was applied to establish allelic association of blood groups and clinical phases according to the area. SPSS 15.0 statistical software and population genetics software were used for calculations.

## 3. Results

As may be seen in Table 1, we sampled 40 individuals in Tomocho-Collicate-Vista Hermosa (prevalence of chronic form), 17 of which (42.5%) were males and 23 were females (57.5%). The average age was 23.5 with a standard deviation of ±10.6. The prevalent clinical phase was the chronic form in 22 cases (57.5%), with only one case of the acute phase (2.5%). CD was not found in 17 (42.5%) of individuals.

The Miraflores group (prevalence of acute form) consisted of 36 individuals, 14 males (38.9%) and 22 females (61.1%). The average age was 29 with a standard deviation of ±22.9. Of these 36 individuals, 12 presented with only acute phase (33.3%), 23 were healthy (63.8%), and one case displayed the chronic and acute phases simultaneously (2.7%).

The biased and compartmentalized distribution of distinct clinical phases in two different but geographically proximal areas, Tomocho-Collicate-Vista Hermosa (prevalence of verrucous phase) and Miraflores (prevalence of hematic phase), promoted us to compare the distribution of phenotype frequencies of the MN, Ss, Diego, and Duffy blood group systems in both areas.

We performed the analysis of the phenotype frequencies of the MN, Ss, Diego, and Duffy blood group systems and found that they were in Hardy-Weinberg equilibrium (Table 2). Thus, at this moment we do not have any evidence of selective pressure on these blood systems. The frequencies of blood group systems were similar in both areas and the MN blood group system was the most variable.

For the MN blood system an increase in the frequency of heterozygotes in Tomocho-Collicate-Vista Hermosa (MM = 0.375, MN = 0.575) was found in comparison to Miraflores (MM = 0.5; MN = 0.389), but it was not statistically significant. However, when the alleles were compared, a much narrower distribution was found, in Tomocho-Collicate-Vista Hermosa the M = 0.6625 and N = 0.3375 versus M = 0.6945 and N = 0.3055 in Miraflores. In both comparisons of distributions of phenotypes and allele frequencies of MN blood group, no significant statistical differences were found between the two areas (Table 2).

In the comparisons of blood group systems Ss, Diego, and Duffy, no evident differences were seen either in phenotypes or alleles. There was, therefore, an absence of statistical differences in all blood group systems with regard to phenotypes and allele frequencies in the two areas (Table 2).

To test the hypothesis that the clinical phases of CD are associated with blood groups, we seek the possible association between phenotypes and alleles of MN, Ss, Diego, and Duffy with the benign (chronic or verrucose) and nonbenign (acute or hematic) clinical phases. Forty individuals were from Tomocho-Collicate-Vista Hermosa districts (prevalence of the verrucose phase) and 36 from Miraflores district (prevalence of the hematic phase). It may be seen, however, that in each group there was one atypical individual for the prevalence of the respective location (one acute case in Tomocho-Collicate-Vista Hermosa and one chronic case in Miraflores). These individuals were removed for subsequent analysis. Therefore, we have selected 39 individuals from Tomocho-Collicate-Vista Hermosa: 22 cases of chronic phase and 17 with no bartonellosis registered and from Miraflores we have 35 individuals: 12 cases of acute phase and 23 with no bartonellosis (Table 3).

The differences of phenotype or alleles among cases versus occurrences of no bartonellosis in each area are nonsignificant statistical. We have also compared the clinical phases and phenotypes or alleles frequencies between areas, and again no significant statistical differences were found (Table 3).

## 4. Discussion and Conclusion

Carrion's disease, a human bartonellosis, is characterized by two well defined clinical phases. One phase is the hematic, anemic, febrile, or acute phase that is devastating and may be fatal and the other is the chronic, eruptive, verrucous phase that is a benign phase [3].

The present and previous work have shown that there is an evident difference of the distribution of the acute and the chronic CD phases in the district of Bagua Grande (MF and JE, not published results, and Annual Report and Epidemiologic Bulletin Subregion of Health Bagua-Amazonas, Peru, 2004). The reason why Tomocho-Collicate-Vista Hermosa displays high prevalence of the chronic phase and Miraflores predominantly displays the acute phase remains without explanation at present. A differential genetic response of the host could be invoked but at this time there is no historical or cultural information concerning the ethnic backgrounds of Miraflores versus Tomocho-Collicate-Villa Hermosa residents. Under the same logic, distinct genotypes-phenotypes linked to a differential distribution of* B. bacilliformis* strains or* Lutzomyia* spp. vector may also be hypothesized, but as yet no proof has been found. Molecular genetic characterization of the pathogen and vector species in bartonellosis needs to be taken into account for future studies concerning the differential distribution in the district of Bagua Grande.

A study regarding the mechanisms of* B. bacilliformis* invasion has revealed the participation of erythrocyte antigens (glycophorins A/B and band 3). Since these molecules are closely related to blood group systems MN, Ss, Diego [15], and Duffy group, it is possible that they could be involved as receptor molecules. All of these proteins are present in the erythrocyte membranes and are involved in the entrance of pathogens like* Plasmodium*. Homozygous defective mutations for these proteins have been reported as being protective for malarial infection in Africa and Asia [19]. Very little information is available from South American populations but some studies in Brazil and Colombia have confirmed that Duffy, MN, and Ss variant blood groups are resistant to* P. falciparum* infection in some populations [20–22], although this concerns the protozoan, studies in bacteria such as* B. bacilliformis* are currently underway.

In our hypothesis, we postulated that selective pressure exerted by this bacterium could have determined different phenotype frequencies of the blood groups from each area under study. Our results indicate no relationship between blood groups and locations that have biased prevalence either with the chronic phase (Tomocho-Collicate-Vista Hermosa) or the acute phase (Miraflores). Phenotypes and allele of blood group systems MN, Ss, Diego, and Duffy do not show a statistically significant (*P* > 0.05) difference between the frequencies found in Tomocho-Collicate-Vista Hermosa compared with the frequencies in Miraflores (Table 2).

Although there was no significant difference (*P* > 0.05) in the MN blood group, which is in Hardy-Weinberg equilibrium in both areas, the incidence of heterozygosity was higher in Tomocho-Collicate-Vista Hermosa than in Miraflores. It is therefore tempting to speculate a heterozygote advantage in Tomocho-Collicate-Vista Hermosa favoring the chronic state. It should however be noted that the nonstatistically relevant differences outlined above could result from the small sample size of this investigation.

The general tendency of no association found with MN and clinical phase continues when we compare Ss, Diego, and Duffy blood group systems alleles with nonaffected individuals and cases of chronic and acute phases (Table 3). However, these data must be considered as preliminary in order to make general inferences about the infection associated with blood group systems or erythrocyte antigens. Other gene variants associated with infectious processes such as genes for toll-like receptors, interleukins, lectins, HLA, cytokines, and other proteins of the innate and acquired immunoinflammatory responses, including microRNA polymorphisms, expression, exomes, and ancestry informative markers (AIMs) need to be tested. Moreover, molecular genetic analysis of bacterium and genetic variability of insect vectors should also be considered [8, 23, 24].

On one hand, the results of this study cannot be extrapolated to other regions of Peru where the disease is also found, since there are differences in the genetic composition of Peruvian subpopulations. The prevalence of the clinical phase and history of outbreaks and reappearances [7] will be very important information when exploring genetic studies amongst these subpopulations. On the other hand, this study has contributed to the knowledge concerning the genetic variability of subpopulations of Bagua Grande, Amazonas.

In conclusion, our results showed no significant statistical differences between allele frequencies of blood groups MNS, Diego, and Duffy and the prevalence of clinical phases of the Carrion's disease in the district of Bagua Grande, department of Amazonas, Peru.

## Figures and Tables

**Table 1:** General data and clinical phases of the samples analyzed in this study.

Characteristics	Tomocho-Collicate-Vista Hermosa	Miraflores
	*n* (%)	*n* (%)
Sample	40	36
Sex		
Male	17 (42.5)	14 (38.9)
Female	23 (57.5)	22 (61.1)
Total	**40 (100.0)**	**36 (100.0)**
Age		
*X* ± SD years	23.5 ± 10.6	29.5 ± 22.9
Clinical phases*		
Acute phase only	1^a^	12
Chronic phase only	22	0
Acute and chronic phases	0	1^b^
No reported disease or healthy	17	23
Total	**40**	**36**
Total for analysis	**39** ^ a^	**35** ^ b^

*X*  ± SD is the mean ± standard deviation.

*Clinical phases' data of each individual were obtained by self-report (personal interviews) and/or review of clinical records for verification of treatment or supervision of the Carrion's disease.

^a^One individual had acute phase and was removed from the analysis “blood group versus clinical phase” in Tomocho-Collicate-Vista Hermosa area, reducing our sample from 40 to 39 individuals.

^b^One individual had acute and chronic phases and was removed from the analysis “blood group versus clinical phase” in Miraflores area, reducing our sample from 36 to 35 individuals.

**Table 2:** Distribution of blood group systems by area, Bagua, Amazonas, Perú.

Group	Phenotypes	Tomocho-Collicate-Vista Hermosa				Miraflores				*P* phenotypes	*P* alleles
		*n* = 40	Phenotype frequency	Allele	Allele frequency	*n* = 36	Phenotype frequency	Allele	Allele frequency		
MN	MM	15	0.375	M	0.6625	18	0.500	M	0.6945	0.232	0.674
	MN	23	0.575	N	0.3375	14	0.389	N	0.3055		
	NN	2	0.050			4	0.111				

Ss	SS	10	0.250	S	0.5625	10	0.278	S	0.5835	0.955	0.796
	Ss	25	0.625	s	0.4375	22	0.611	s	0.4165		
	Ss	5	0.125			4	0.111				

Diego	Di (a+)	6	0.150	Di a	0.0750	7	0.194	Di a	0.0972	0.607	0.625
	Di (a−)	34	0.850	No Di a	0.9250	29	0.806	No Di a	0.9027		

Duffy	Fy (a+b−)	14	0.350	Fy a	0.5750	13	0.361	Fy a	0.5695	0.952	0.945
	Fy (a+ b+)	18	0.450	Fy b	0.4250	15	0.417	Fy b	0.4305		
	Fy (a− b+)	8	0.200			8	0.222				
	Fy (a− b−)	0	0.000			0	0.000				

Phenotype frequencies (in this case also genotype) of MN blood group system, the most variable, are in Hardy-Weinberg equilibrium (*P* > 0.05, Chi-square test). In the comparisons of phenotypes and allele frequencies of blood group systems, no significant statistical differences (*P* > 0.05, Chi-square or Fisher's exact tests) were found between the two areas.

**Table 3:** Phenotypes and allele frequencies of blood group systems according clinical phases by area study, Bagua, Amazonas, Peru.

Group		Area						*P* ^b^ clinical phases between areas
		Tomocho-Collicate-Vista Hermosa (*n* = 39)*			Miraflores (*n* = 35)*			
		Cases in chronic phase only (*n* = 22)	No reported disease (*n* = 17)	*P* ^a^	Cases in acute phase only (*n* = 12)	No reported disease (*n* = 23)	*P* ^a^	
MN	MM	8	7	0.759	5	12	0.555	0.761
	MN	12	10		6	8		
	NN	2	0		1	3		
	M	28 (0.6364)	24 (0.7059)	0.518	16 (0.6667)	32 (0.6957)	0.804	0.803
	N	16 (0.3636)	10 (0.2941)		8 (0.3333)	14 (0.3043)		

Ss	SS	6	4	0.791	3	6	0.944	0.886
	Ss	14	10		8	14		
	ss	2	3		1	3		
	S	26 (0.5910)	18 (0.5294)	0.587	14 (05834)	26 (0.5653)	0.884	0.952
	s	18 (0.4090)	16 (0.4706)		10 (0.4166)	20 (0.4347)		

Diego	Di (a+)	4	2	0.679	2	5	1.000	1.000
	Di (a−)	18	15		10	18		
	Di a	4 (0.0909)	2 (0.0588)	0.691	2 (0.0833)	5 (0.1086)	1.000	1.000
	No Di a	40 (0.9091)	32 (0.9412)		22 (0.9167)	41 (0.8914)		

Duffy	Fy (a+ b−)	8	6	0.945	4	9	0.736	0.850
	Fy (a+ b+)	9	8		6	9		
	Fy (a− b+)	5	3		2	5		
	Fy (a− b−)	0	0		0	0		
	Fy a	25 (0.5692)	20 (0.5883)	0.859	14 (0.5334)	27 (0.5870)	0.977	0.904
	Fy b	19 (0.4318)	14 (04117)		10 (0.4166)	19 (0.4130)		

*One individual of the Tomocho-Collicate-Vista Hermosa had acute phase and was removed from this analysis, reducing our sample from 40 to 39 individuals. In a similar way, one individual from Miraflores presented chronic phase and was also removed from the analysis, reducing the number from 36 to 35 individuals.

^a^In the comparisons of phenotypes and allele frequencies, considering clinical phases and healthy individuals (2 × 2 clusters), no significant statistical differences (*P* > 0.05, Chi-square or Fisher's exact tests) were found in each area.

^b^In the comparisons of phenotypes and allele frequencies, considering only clinical phases (2 × 2 clusters), no significant statistical differences (*P* > 0.05, Chi-square or Fisher's exact tests) were found between the two areas.
